# Molecular determinants for enzalutamide-induced transcription in prostate cancer

**DOI:** 10.1093/nar/gkz790

**Published:** 2019-09-10

**Authors:** Fuwen Yuan, William Hankey, Dayong Wu, Hongyan Wang, Jason Somarelli, Andrew J Armstrong, Jiaoti Huang, Zhong Chen, Qianben Wang

**Affiliations:** 1 Department of Pathology, Duke University School of Medicine, Durham, NC 27710, USA; 2 Department of Cancer Biology and Genetics, The Ohio State University College of Medicine, Columbus, OH 43210, USA; 3 Department of Medicine, Duke University School of Medicine, Durham, NC 27710, USA; 4 Duke Cancer Institute Center for Prostate and Urologic Cancers, Duke University School of Medicine, Durham, NC 27710, USA; 5 Departments of Surgery, Pharmacology, and Cancer Biology, Duke University School of Medicine, Durham, NC 27710, USA

## Abstract

Enzalutamide, a second-generation androgen receptor (AR) antagonist, has demonstrated clinical benefit in men with prostate cancer. However, it only provides a temporary response and modest increase in survival, indicating a rapid evolution of resistance. Previous studies suggest that enzalutamide may function as a partial transcriptional agonist, but the underlying mechanisms for enzalutamide-induced transcription remain poorly understood. Here, we show that enzalutamide stimulates expression of a novel subset of genes distinct from androgen-responsive genes. Treatment of prostate cancer cells with enzalutamide enhances recruitment of pioneer factor GATA2, AR, Mediator subunits MED1 and MED14, and RNA Pol II to regulatory elements of enzalutamide-responsive genes. Mechanistically, GATA2 globally directs enzalutamide-induced transcription by facilitating AR, Mediator and Pol II loading to enzalutamide-responsive gene loci. Importantly, the GATA2 inhibitor K7174 inhibits enzalutamide-induced transcription by decreasing binding of the GATA2/AR/Mediator/Pol II transcriptional complex, contributing to sensitization of prostate cancer cells to enzalutamide treatment. Our findings provide mechanistic insight into the future combination of GATA2 inhibitors and enzalutamide for improved AR-targeted therapy.

## INTRODUCTION

Lipophilic ligands (e.g. steroids), functioning through nuclear hormone receptors (NRs), play important roles in various physiological processes including sexual maturation, metabolism, immune response and development (1,2). Liganded NRs also regulate many pathological processes such as cancer, inflammation, cardiovascular disease and reproductive disease, making them attractive targets for drug development (3,4). Androgen receptor (AR), a member of the NR superfamily, plays a key role in the onset and progression of prostate cancer (5,6), and numerous synthetic AR antagonists have been developed to inhibit the action of endogenous AR ligands (i.e. androgens) (7,8). A prominent example is enzalutamide (Xtandi®), a second-generation AR antagonist showing robust anti-cancer activity with an expanding application to patient care for both castration-resistant prostate cancer (CRPC) and hormone sensitive prostate cancer (HSPC) (9,10). However, resistance to enzalutamide emerges, subsequently leading to treatment failure (11–14). Thus, the therapeutic efficacy of enzalutamide needs to be improved. Unfortunately, mechanisms underlying the emergence of resistance are largely unknown.

AR is a ligand-induced transcription factor that contains an N-terminal domain (NTD) and a central DNA binding domain (DBD) that is connected by a hinge to the C-terminal ligand-binding domain (LBD) (2). AR regulates target gene expression through binding to androgen responsive elements (AREs) in the presence of androgens (2,15). Enzalutamide competes with androgens to bind AR, and thus inhibits AR binding to AREs and androgen-regulated transcription (9,16). Using a high-resolution ChIP-exo approach, we recently found that enzalutamide induces AR binding to the novel binding motif 5′-NCHKGNnndDCHDGN, stimulating the expression of several antagonist-responsive, cancer-relevant genes (e.g. *CPEB4*) in prostate cancer cells (16). Thus, enzalutamide may act as a partial transcriptional agonist. However, it is unknown to what extent enzalutamide modulates global transcription. More importantly, the molecular mechanisms underlying enzalutamide-mediated transcription have not been completely elucidated.

In this study, we find that enzalutamide stimulates expression of a novel class of genes that are not regulated by the physiological androgen, dihydrotestosterone (DHT). We further demonstrate that the pioneer factor GATA2 plays a critical role in globally regulating enzalutamide-induced transcription by recruiting AR, Mediator, and RNA Pol II (Pol II) to antagonist-responsive gene loci. Importantly, our study demonstrates that the GATA2 inhibitor K7174 perturbs enzalutamide-liganded AR-mediated transcription activation and markedly enhances the ability of enzalutamide to decrease prostate cancer cell proliferation. These results reveal molecular mechanisms underlying enzalutamide-induced transcription and provide a potential combined therapy strategy for more effectively inhibiting AR signaling in prostate cancer.

## MATERIALS AND METHODS

### Reagents and antibodies

All chemicals and reagents were purchased either from Sigma-Aldrich (Merck) or Thermo Fisher Scientific (Invitrogen). Commercial antibodies used for ChIP and western blotting were purchased from the following companies: Anti-AR for ChIP (Abcam, ab74272), Anti-GATA2 for ChIP and western blotting (Santa Cruz, sc9008), Anti-FOXA1 for ChIP (Abcam, ab23738), Anti-RNA Pol II for ChIP (Abcam, ab5408), Anti-MED1 for ChIP and western blotting (Santa Cruz, sc8998x); Anti-MED14 for ChIP and western blotting (Fisher Scientific, PIPA544864). Anti-Calnexin for western blotting (ENZO, AD1-SPA-860). Secondary antibodies used for western blotting, HRP Goat anti-Mouse (926-80010) and HRP Goat anti-Rabbit (926-80011), were purchased from LI-COR Biosciences.

### Cell culture and transfection

LNCaP and LAPC4 cells were obtained from the American Type Culture Collection and were cultured in RPMI1640 with 10% FBS or MEM with 10% FBS, respectively. CWR22Rv1 cells were obtained from the American Type Culture Collection (ATCC) and were cultured in DMEM with 10% FBS. For ChIP experiments, cells were maintained in phenol red-free medium with 5% charcoal-stripped FBS for 3 days, and then treated with vehicle and different ligands. For quantitative RT-PCR assays, cells were maintained in phenol red-free medium with 10% charcoal-stripped FBS for 2 days, and then treated with vehicle and different reagents. All transient transfections of siRNA followed the standard protocol of Lipofectamine RNAiMAX Transfection Reagent (Thermo Fisher). Further details are as described previously (17). siRNAs used in this study are listed in Supplementary Table S1.

### RNA-seq and data analysis

RNA-seq analysis was performed and analyzed as previously described with minor modifications (18). LNCaP cells were treated with 10 nM DHT or 10 μM enzalutamide for 4 and 24 h, respectively. RNA was extracted using the RNeasy Mini Kit (Qiagen). mRNA was enriched using NEBNext Poly(A) mRNA Magnetic Isolation Module. After two rounds of processing, the mRNA was recovered for library generation. The library preparation was performed with NEBNext^®^ Ultra™ Directional RNA Library Prep Kit for Illumina following the manufacturer's instructions. The cDNA molecules were amplified by 8 cycles of PCR. Non-size selected libraries were then sequenced using Illumina HiSeq 2500 at the Ohio State University Comprehensive Cancer Center sequencing core. Read alignment was conducted using TopHat 2.0.13, and relative transcript abundances and differentially expressed genes were determined using Partek Genomics Suite (v6.6) with default settings. Gene Ontology (GO) analysis of enzalutamide- and DHT-regulated genes was performed using Genomatix Pathway System (v3.3). To assess the impact of GATA2 silencing on enzalutamide-induced transcription, LNCaP cells were transfected with *GATA2* siRNA pool (Dharmacon, ON-TARGETplus Human GATA2 siRNA SMARTpool) or a control siRNA pool (Dharmacon, ON-TARGETplus Non-targeting SMARTpool). Seventy-two h posttransfection, cells were treated with 25 μM enzalutamide or vehicle for twenty-four h, and RNA-seq analysis was conducted as described above. Libraries were sequenced using Illumina HiSeq 4000 at Duke Sequencing and Genomic Technologies shared resource. Enzalutamide-upregulated genes (>2-fold) are listed in Supplementary Tables S2 and S3.

### Standard ChIP assays

ChIP assays were performed as described previously (19). Briefly, cells were crosslinked with 1% formaldehyde for 10 min at room temperature and chromatin was collected, sonicated, diluted and immunoprecipitated with 4 μg of specific antibodies at 4°C overnight. Protein A-Sepharose beads were added and incubated for another 1 h with rotation. The beads were then washed sequentially for 10 min each in TSE I (0.1% SDS, 1% Triton X-100, 2 mM EDTA, 20 mM Tris–HCl, pH 8.1, 150 mM NaCl), TSE II (0.1% SDS, 1% Triton X-100, 2 mM EDTA, 20 mM Tris–HCl, pH 8.1, 500 mM NaCl), and buffer III (0.25 M LiCl, 1% NP-40, 1% deoxycholate, 1 mM EDTA, 10 mM Tris–HCl, pH 8.1) and finally twice with TE buffer. Chromatin complexes were eluted with elution buffer (1% SDS, 0.1 M NaHCO_3_) and de-crosslinked at 65°C overnight. DNA fragments were purified with the QIAquick PCR purification kit (Qiagen 28104) and used for quantitative PCR reactions with Power SYBR Green PCR Master Mix reagents (Applied Biosystems). Primers used for ChIP are listed in Supplementary Table S4.

### Quantitative RT-PCR

Quantitative RT-PCR was performed as previously described (20). Briefly, cells were treated with vehicle, enzalutamide or K7174 or transfected with siRNA and cultured for the indicated time, then total RNA was isolated with the RNeasy Mini kit (Qiagen, 74104). qRT-PCR was conducted using the MultiScribe Reverse Transcriptase and Power SYBR Green PCR Master Mix reagents (Applied Biosystems), according to the manufacturer's instructions. Each assay was repeated three to four times. Primers used are listed in Supplementary Table S5.

### Western blotting assays

Western blotting was performed as previously described (20). Briefly, cells were collected and lysed in RIPA buffer (1% NP-40, 0.1% sodium dodecyl sulfate (SDS), 50 mM Tris–HCl pH 7.4, 150 mM NaCl, 0.5% sodium deoxycholate, 1 mM ethylenediaminetetraacetic acid (EDTA), 1× proteinase inhibitor cocktail (Roche)) for 20 min on ice and the proteins were resolved on 8% SDS-polyacrylamide gels and transferred onto Nitrocellulose membrane (Bio-Rad). The membrane was blocked with 5% milk powder (Bio-Rad) then incubated with specific antibodies at 4°C overnight. Following incubation with secondary antibodies, immunoblots were visualized using the C-DiGit Chemiluminescent Western Blot Scanner (Li-Cor). Antibodies used for western blotting are listed in ‘Reagents and Antibodies’.

### Cell proliferation assays

Cell proliferation was quantified by WST-1 assays and BrdU incorporation assays. LNCaP cells or LAPC4 cells were seeded in 96-well plates (2 × 10^4^ or 5 × 10^4^ cells/ml; 100 μl/well). The cells were treated with vehicle or 10 μM K7174 together with different doses of enzalutamide and cultured in complete medium with 10% FBS. For WST-1 assays, the commercially available Cell Proliferation Reagent WST-1 (11644807001, Sigma-Aldrich) was used for measuring cell proliferation.WST-1 was added to each well (10 μl/well) at 1 day, 3 days and 5 days after cell seeding. After 1 h of incubation, absorbance at 450 nM was measured using a microplate reader (SpectraMax M3, Molecular Devices, Inc., Sunnyvale, CA, USA). BrdU incorporation was measured by a commercial BrdU cell proliferation assay kit (Cell Signaling Technology, Inc., Danvers, MA) according to the manufacturer's protocol after cells were treated with the indicated drugs for 3 days. Both methods were repeated for each cell line (*n* = 6).

### Quantitative chromosome conformation capture assay

Quantitative chromosome conformation capture (3C) qPCR assays were performed as described (21) with some modifications. Briefly, nuclei were cross-linked with formaldehyde at a final concentration of 1% for 10 min, then digested with 400 units of HindIII (NEB) and ligated under extra-diluted conditions. After reversal of cross-linking, DNA was purified by phenol–chloroform extraction followed by ethanol precipitation. Real-time PCR was performed using TaqMan^®^ Universal PCR Master Mix (Applied Biosystems). The probe and primer sequences are listed in Supplementary Table S6. BAC RP11-815F2 constructs containing DNA fragments covering the tested regions were used as template controls for normalizing digestion, ligation and primer efficiency. *GADPH* loading control was used to normalize DNA concentration. The interaction of two HindIII sites in the *GADPH* locus was used for comparison between different 3C assays.

### CRISPR/Cas9-mediated deletion of the enhancer region

CRISPR/Cas9-mediated deletion of the enhancer regions in the *NR3C1* and *SLC7A11* loci was conducted as described before (22) with some modifications. CRISPR/Cas9 sgRNAs were identified using the MIT CRISPR Design tool, and non-targeting sgRNAs were used as reported in Zhang *et al.* (22). All sgRNA sequences are listed in Supplementary Table S7. Tandem U6 promoter–sgRNA and H1 promoter–sgRNA cassettes were cloned into lentiCRISPR_v2 (Addgene, 60954) for single-vector expression of two sgRNAs. After infection, cells were selected with 2 μg/ml puromycin. To detect deletion of the enhancer, genomic DNA was first extracted and used for PCR with Q5 high-fidelity DNA polymerase (NEB, M0491) together with the primers listed in Supplementary Table S7.

### Statistics

Statistical significance was evaluated using two-sided unpaired *t*-tests. In the figures with bar graphs, values are expressed as the mean ± S.D. *P*-values ≤ 0.05 are considered statistically significant: ** indicates *P* ≤ 0.01; * indicates *P* ≤ 0.05.

## RESULTS

### Enzalutamide- but not DHT-liganded AR regulates the *NR3C1* and *SLC7A11* genes

**Figure 1.**
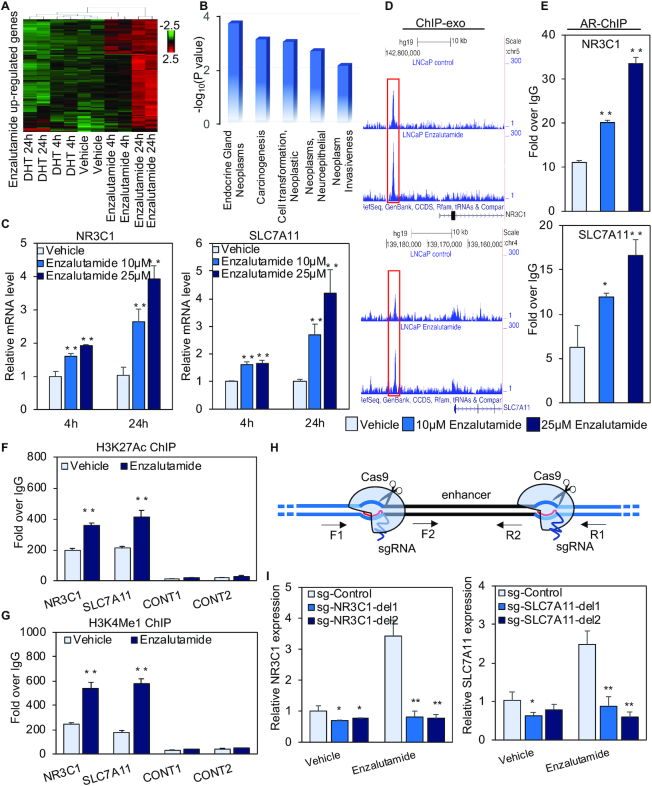
Enzalutamide regulates global, cancer-relevant transcription. (**A**) A heatmap of enzalutamide upregulated genes. The gene expression (reads per kb per million mapped reads (RPKM)) values for each gene were normalized to the standard normal distribution to generate *Z*-scores. The scale bar is shown with the minimum expression value for each gene in green and the maximum value in red. (**B**) Genomatix Pathway containing enzalutamide upregulated genes. (**C**) Expression of *NR3C1* and *SLC7A11* was determined by quantitative RT-PCR after LNCaP cells were treated with vehicle or 10 μM or 25 μM enzalutamide for 4 or 24 h. (**D**) IGV shows AR ChIP-exo data at the *NR3C1* and *SLC7A11* loci. (**E**) AR ChIP validation of the *NR3C1* and *SLC7A11* loci in LNCaP cells treated with vehicle or 10 μM or 25 μM enzalutamide for 4 h. (F and G) The enrichment of H3K4Me1 (**F**) and H3K27Ac (**G**) within the *NR3C1* and *SLC7A11* loci in LNCaP cells treated with vehicle or with 25 μM enzalutamide for 4 h was determined by ChIP assays. (**H**) Schematic of CRISPR/Cas9-mediated deletion of the enhancers. The annealing sites for the primers used to validate deletion are indicated. (**I**) Expression of *NR3C1* and *SLC7A11* was measured by quantitative RT-PCR after cells were treated with vehicle or 25 μM enzalutamide for 24 h. sg-Control, a pair of sgRNAs that are predicted to not recognize any genomic regions; sg-del1 and sg-del2, two separate pairs of sgRNAs recognizing the boundaries of the enhancer region. RNA-seq was performed with two biological replicates (*r* > 0.99, Spearman's correlation). Statistical significance was assessed using the Student's *t*-test, *P < 0.05, **P < 0.01. Results were reported as the mean of two to four replicates, with error bars representing the standard deviation.

Our previous study found that enzalutamide-liganded AR directly upregulates several target genes (e.g*. CPEB4*) (16). This motivated us to investigate the extent to which enzalutamide regulates transcription on a global scale. LNCaP cells were treated with 10 μM enzalutamide or 10 nM DHT for 4 h and 24 h, and RNA-seq assays were performed as we described previously (18). In two biological replicates with high reproducibility (*r* > 0.99, Spearman's correlation), we found that the vast majority (91.2%) of enzalutamide upregulated genes were not regulated by DHT (Figure 1A, Supplementary Figure S1A, B, and Supplementary Table S2). Among genes stimulated by enzalutamide were those involved in endocrine gland neoplasm, carcinogenesis and neoplastic cell transformation (Figure 1B), whereas genes regulated by DHT were associated with canonical prostate cancer-related processes (Supplementary Figure S2A–C). We next performed pathway analysis to study the functional and physical interactions among the enzalutamide-stimulated genes. This identified *NR3C1* (*GR*) as the key hub gene of the enzalutamide-stimulated gene network (Supplementary Figure S1C). To the best of our knowledge this is the first study demonstrating that short-term enzalutamide treatment (4 and 24 h) stimulates *NR3C1* expression, although increased *NR3C1* expression was reported in long-term treated enzalutamide-resistant LNCaP cells genetically engineered to overexpress AR (23). We next validated the RNA-seq data by performing RT-PCR analysis of the *NR3C1* gene. As the *SLC7A11* gene within the enzalutamide-stimulated gene network (Supplementary Figure S1C) plays an important role in promoting cancer growth and metastasis (24–26), we also validated *SLC7A11* expression. Enzalutamide (10 μM) but not DHT (10 nM) treatment for 4 or 24 h significantly increased mRNA expression of *NR3C1* and *SLC7A11* (Figure 1C and Supplementary Figure S3A). Enzalutamide treatment also enhanced protein expression of NR3C1 and SLC7A11 (Supplementary Figure S3B). Given that patients taking 160 mg/day enzalutamide (current recommended dose) have a mean minimum blood serum concentration of enzalutamide of about 24.55 μM, and that the maximum blood serum concentration is about 1.25× higher (27,28), we next treated the cells with 25 μM enzalutamide to better imitate the real dose of enzalutamide in patients. Interestingly, 25 μM enzalutamide treatment resulted in higher expression levels of *NR3C1* and *SLC7A11* compared with 10 μM enzalutamide treatment (Figure 1C). In contrast, neither 10 μM nor 25 μM enzalutamide treatment stimulated expression of two canonical target genes of DHT-liganded AR, *PSA* and *TMPRSS2* (Supplementary Figure S3C). To support these findings, we performed RT-PCR analysis in two additional prostate cancer cell lines, LAPC4 and CWR22Rv1. Similarly, enzalutamide stimulated transcription of *NR3C1* and *SLC7A11* but not *PSA* or *TMPRSS2* (Supplementary Figure S3D and E), suggesting that enzalutamide-stimulated transcription is not limited to a single prostate cancer cell line. To determine whether other AR antagonists are able to induce transcription of *NR3C1* and *SLC7A11*, we measured expression of *NR3C1* and *SLC7A11* after LNCaP cells were treated with bicalutamide, apalutamide or darolutamide. Bicalutamide and darolutamide but not apalutamide treatment significantly upregulated *NR3C1* and *SLC7A11* expression (Supplementary Figure S4), demonstrating that AR antagonist-induced transcription in prostate cancer cells is not limited to a single AR antagonist.

We next searched our AR ChIP-exo datasets (16) and identified enzalutamide-induced AR binding sites –13.9 and –12.8 kb from the transcription start sites (TSS) of the *NR3C1* and *SLC7A11* genes, respectively (Figure 1D). Validation of ChIP-exo results by standard ChIP assays in LNCaP cells treated with 10 or 25 μM enzalutamide found that enzalutamide treatment significantly increased AR binding to regulatory regions of the *NR3C1* and *SLC7A11* genes in a dose-dependent manner (Figure 1E). In contrast, 10 or 25 μM enzalutamide treatment failed to promote AR binding to the *PSA* and *TMPRSS2* enhancers (Supplementary Figure S3F). Together, these data suggest that enzalutamide dose-dependently increases AR binding to regulatory elements of two novel AR target genes, *NR3C1* and *SLC7A11*. Our subsequent experiments were performed using cells treated with enzalutamide at the clinically relevant dose of 25 μM. To investigate whether the distal regulatory regions of the *NR3C1* and *SLC7A11* genes are functional, we first determined the levels of histone modifications H3K4me1 (histone H3 lysine 4 monomethylation) and H3K27Ac (histone H3 lysine 27 acetylation) within these regions. H3K4me1 and H3K27Ac were enriched within *NR3C1* and *SLC7A11* regulatory elements and the enrichment was further enhanced by enzalutamide treatment (Figure 1F and G), supporting an active enhancer role of these two distal elements (29). Importantly, CRISPR/Cas9-based deletion of these distal elements that loop to the promoter abolished enzalutamide-induced transcription (Supplementary Figure S5A, B and Figure 1H, I). These data demonstrate that the *NR3C1* and *SLC7A11* enhancers respectively mediate enzalutamide-stimulated expression of these two genes.

### Pioneer factor GATA2 globally governs enzalutamide-induced transcription

**Figure 2.**
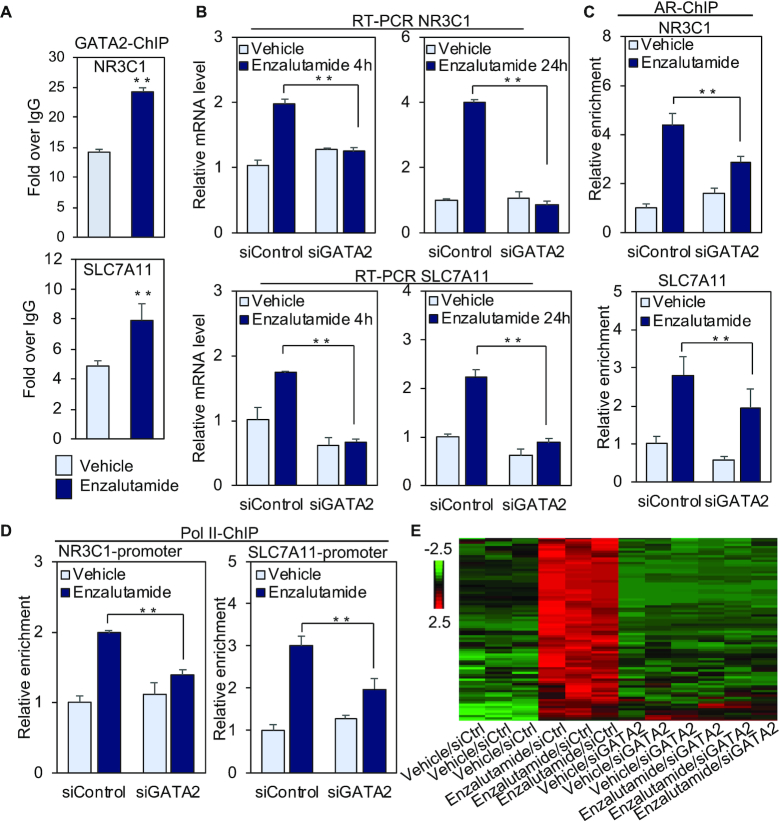
GATA2 promotes enzalutamide-induced gene transcription by recruiting AR and RNA Pol II. (**A**) LNCaP cells were treated with vehicle or 25 μM enzalutamide for 4 h, and GATA2 ChIP was performed on distal *NR3C1* and *SLC7A11* regulatory regions. (**B**) LNCaP cells were transfected with *GATA2* siRNA or control siRNA, and cells were treated with vehicle or 25 μM enzalutamide for 4 h or 24 h. Total RNA was isolated and amplified with gene-specific primers. (C and D) LNCaP cells were transfected with *GATA2* siRNA or control siRNA, and cells were exposed to vehicle or 25 μM enzalutamide for 4 h. AR ChIP assays (**C**) were performed on distal *NR3C1* and *SLC7A11* regulatory regions, and Pol II ChIP (**D**) assays were conducted on the *NR3C1* and *SLC7A11* promoters. (**E**) A heatmap of genes upregulated by 25 μM enzalutamide. The gene expression (reads per kb per million mapped reads (RPKM)) values for each gene were normalized to the standard normal distribution to generate *Z*-scores. The scale bar is shown with the minimum expression value for each gene in green and the maximum value in red. Statistical significance was assessed using the Student's *t*-test, **P < 0.01, and results were reported as the mean of two to four replicates, with error bars representing the standard deviation.

We next investigated the underlying mechanisms for enzalutamide-liganded AR regulation of *NR3C1* and *SLC7A11*. Given that the pioneer transcription factors GATA2 and FOXA1 affect AR regulation of DHT-responsive genes (21,30), and that GATA2 (Supplementary Figure S6) and FOXA1 (16) bind to enzalutamide-responsive AR locations, we performed ChIP assays to confirm the binding of GATA2 or FOXA1 to the *NR3C1* and *SLC7A1* enhancers. Both GATA2 and FOXA1 were enriched at these two enhancer regions, and enzalutamide treatment further enhanced the recruitment of these two pioneer factors (Figure 2A and Supplementary Figure S7A). To investigate whether binding of GATA2 or FOXA1 regulates *NR3C1* and *SLC7A11*, we knocked down *GATA2* or *FOXA1*, followed by RT-PCR analyses of these two genes in LNCaP cells treated with or without enzalutamide for 4 h and 24 h. The siRNA knockdown efficiency was confirmed by RT-PCR and western blot analyses (Supplementary Figure S7B and C). *GATA2* silencing uniformly and significantly inhibited enzalutamide-induced expression of *NR3C1* and *SLC7A11* at both 4 and 24 h time points (Figure 2B). In contrast, *FOXA1* knockdown enhanced or inhibited enzalutamide-stimulated expression at different time points in a gene-specific manner (Supplementary Figure S7D). This is consistent with the reported complex role of FOXA1 in the regulation of androgen-responsive genes (31–33). These data suggest that GATA2 plays a general role in mediating activation of *NR3C1* and *SLC7A11* by enzalutamide.

To address the underlying mechanisms of GATA2 regulation of enzalutamide-induced transcription, we performed ChIP assays to study the effect of *GATA2* silencing on AR and Pol II recruitment to regulatory regions of *NR3C1* and *SLC7A11*. Recruitment of AR to the enhancers and of Pol II to the promoters was significantly decreased in siGATA2 transfected cells compared with the siControl in the presence of enzalutamide (Figure 2C and D). These data suggest that GATA2 is required for enzalutamide-induced AR and Pol II recruitment to *NR3C1* and *SLC7A11* loci.

To investigate whether GATA2 globally regulates enzalutamide-induced transcription, RNA-seq assays were performed in siControl- or siGATA2 transfected LNCaP cells treated with 25 μM enzalutamide or vehicle for 24 h. Interestingly, we found that the vast majority of (94.04%) enzalutamide-upregulated genes were downregulated by GATA2 knockdown (Figure 2E, Supplementary Figure S8 and Supplementary Table S3), suggesting that GATA2 directs enzalutamide-induced global transcriptional activation. Further integrative analysis of enzalutamide-liganded AR binding locations (16) and enzalutamide/GATA2-regulated genes revealed that enzalutamide-liganded AR binding locations were located at non-promoter regions of these genes (Supplementary Figure S9). To validate our global analysis, RT-PCR was applied to analyze 4 novel enzalutamide regulated genes (*TSC22D3, LAMP3, VEGFA* and *CEMIP*). Expression of each of these four genes was upregulated by enzalutamide but not DHT, and GATA2 silencing significantly decreased enzalutamide-stimulated expression (Supplementary Figure S10A and B). In addition, enzalutamide-stimulated binding of GATA2 and AR to distal regulatory regions of these four genes was significantly inhibited after GATA2 knockdown (Supplementary Figure S10C and D). Taken together, these results suggest that GATA2 governs enzalutamide-induced global transcription. Finally, we examined the functional significance of our newly-discovered enzalutamide-activated genes, *NR3C1, SLC7A11, TSC22D3, LAMP3, VEGFA* and *CEMIP*. Silencing of each of these genes significantly enhanced the cell growth inhibitory effect of enzalutamide (Supplementary Figure S11), suggesting that expression of these genes compromises the ability of enzalutamide to inhibit prostate cancer growth.

**Figure 3.**
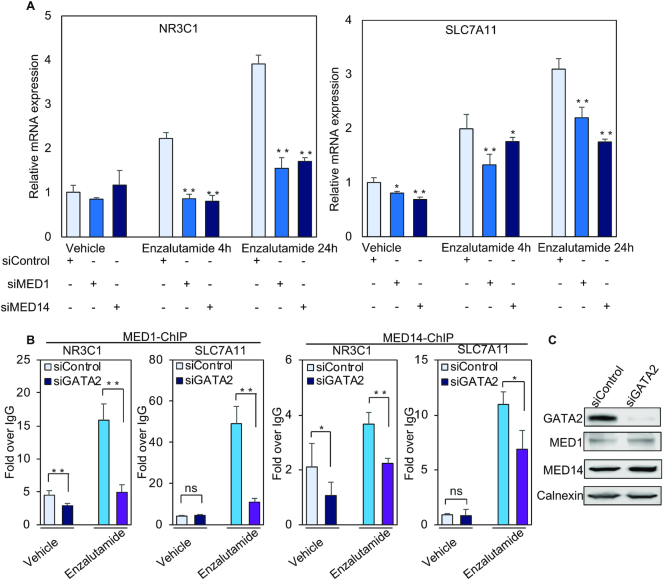
MED1 and MED14 coactivate enzalutamide-induced transcription. (**A**) Total RNA was isolated to analyze the relative expression of representative genes using quantitative RT-PCR after LNCaP cells were transfected with control siRNA or *MED1* or *MED14* siRNA pools and then treated with vehicle or 25 μM enzalutamide for the indicated time. (**B**) The enrichment of MED1 and MED14 on the enhancers of *NR3C1* and *SLC7A11* was analyzed with ChIP assays using the MED1 antibody and MED14 antibody after LNCaP cells were transfected with control siRNA or *GATA2*. (**C**) The protein expression of MED1 and MED14 was determined by western blot after LNCaP cells were transfected with *GATA2* siRNA pool. ns, not significant, *P < 0.05, **P < 0.01. Results of ChIP and quantitative RT-PCR assays reported as the mean of two to four replicates, with error bars representing the standard deviation.

Given that transcription coactivators play important roles in androgen-induced transcription (34,35), we next asked if coactivators are involved in enzalutamide-regulated transcription. Our previous studies have found that the coactivator MED1, a subunit of an evolutionarily conserved multiprotein complex (∼30 subunits) known as the Mediator, is essential for androgen-regulated transcription in prostate cancer cells (35,36). We thus examined the role of MED1 in enzalutamide-induced activation of *NR3C1, SLC7A11, TSC22D3, LAMP3, VEGFA* and *CEMIP*. Given that the MED14 subunit in particular was recently identified as a structural and functional backbone of the Mediator complex (37,38), we also investigated whether MED14 is involved in enzalutamide-stimulated transcription. Forty-eight hours after *MED1* or *MED14* siRNA transfection (Supplementary Figure S12A and B), cells were treated with enzalutamide for 4 h or 24 h. Silencing of *MED*1 or *MED14* significantly decreased enzalutamide-induced transcription of the six genes at 4 h and/or 24 h time points (Figure 3A and Supplementary Figure S12C), suggesting that MED1 and MED14 coactivate enzalutamide-stimulated transcription. Since GATA2 is reported to recruit MED1 to regulatory regions of some androgen-responsive genes (21), we next examined whether GATA2 facilitates recruitment of MED1 and MED14 to the enhancer of *NR3C1* and *SLC7A11*. *GATA2* silencing significantly attenuated enzalutamide-stimulated MED1 and MED14 binding to both of these enhancers (Figure 3B) without affecting MED1 or MED14 protein expression levels (Figure 3C). These data suggest that GATA2 directs MED1 and MED14 to coactivate enzalutamide-stimulated transcription. Consistent with the known role of Mediator to facilitate Pol II recruitment to the transcription preinitation complex (PIC) (39), our results also indicate that GATA2 directs Pol II binding (Figure 2D) through the recruitment of MED1 and MED14.

### GATA2 inhibitor K7174 impairs enzalutamide-responsive transcription and prostate cancer growth

**Figure 4.**
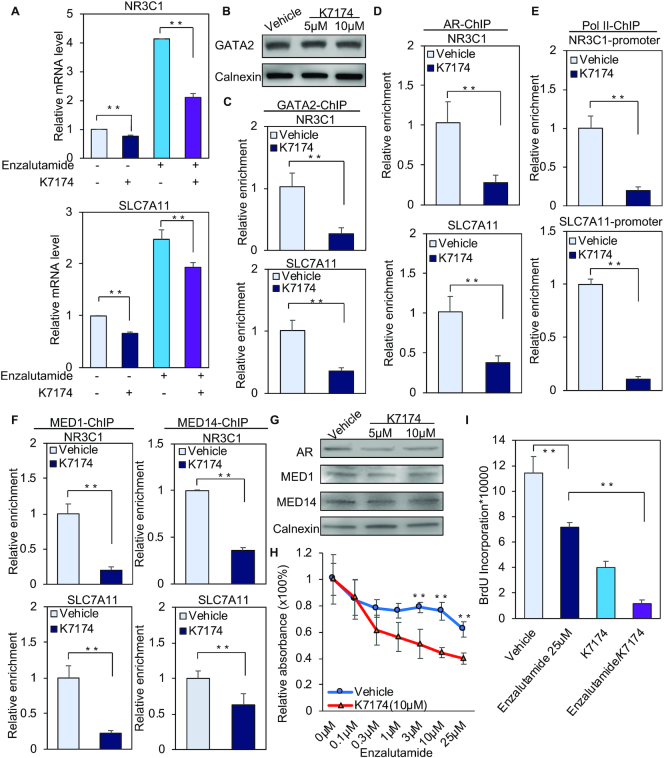
GATA2 inhibitor K7174 attenuates enzalutamide-induced gene transcription and represses prostate cancer cell proliferation. (**A**) LNCaP cells were treated for 24 h with vehicle, 25 μM enzalutamide, 10 μM K7174 or 25 μM enzalutamide together with 10 μM K7174. RT-PCR was performed with the total RNA. (**B**) Western blot analysis of GATA2 protein levels after LNCaP cells were treated with vehicle or the indicated dose of K7174 for 24 h. (C–F) LNCaP cells were treated with vehicle or 10 μM K7174 for 20 h, then with vehicle or 25 μM enzalutamide for another 4 h. GATA2 ChIP assays (**C**), AR ChIP assays (**D**), or MED1 and MED14 ChIP assays (**E**) were performed on *NR3C1* and *SLC7A11* enhancers, and Pol II ChIP (**F**) assays were conducted on the *NR3C1* and *SLC7A11* promoters. **(G)** The protein expression of AR, MED1 and MED14 was detected by western blot after the LNCaP cells were treated with vehicle or 5 μM K7174 or 10 μM K7174 for 24 h. (H and I) LNCaP cells were split in 96 well plates (5,000 cells per well) and were treated with vehicle or 10 μM K7174 together with different concentrations of enzalutamide for 72 h. WST-1 assay (**H**) and BrdU incorporation assay (**I**) were performed to analyze the relative cell proliferation. **P < 0.01. Results of ChIP and quantitative RT-PCR assays are reported as the mean of two to four replicates, with error bars representing the standard deviation.

Given that GATA2 can be targeted with the small molecule inhibitor K7174 (40,41), we next tested if K7174 treatment impairs enzalutamide-induced transcription and prostate cancer growth. Treatment with 10 μM K7174 significantly inhibited enzalutamide-induced expression of *NR3C1, SLC7A11, TSC22D3, LAMP3, VEGFA* and *CEMIP* in LNCaP (Figure 4A and Supplementary Figure S13). Similar results were obtained in LAPC4 cells (Supplementary Figure S14A). While K7174 treatment did not affect the GATA2 protein level (Figure 4B and Supplementary Figure S14B), it significantly decreased GATA2 binding to regulatory regions of *NR3C1* and *SLC7A11* (Figure 4C). We next examined the effect of K7174 on recruitment of AR, Pol II, MED1 and MED14 to regulatory elements of *NR3C1, SLC7A11, TSC22D3, LAMP3, VEGFA* and *CEMIP*. Exposure to K7174 significantly attenuated the recruitment of AR, coactivators and Pol II to regulatory regions of these six genes (Figure 4D–F and Supplementary Figure S15). Similar to the case of GATA2, exposure to K7174 had no effect on protein expression levels of AR or Mediator (Figure 4G). Taken together, these data suggest that K7174 decreases enzalutamide-induced transcription complex loading on regulatory regions of enzalutamide-activated genes. This may account, at least in part, for the decreased mRNA expression of these genes after K7174 treatment.

Finally, we examined whether K7174 affects enzalutamide-inhibited cell growth. Importantly, exposure to K7174 sensitized LNCaP and LAPC4 cells to enzalutamide treatment (Figure 4H, I and Supplementary Figure S16), suggesting that K7174 inhibition of transcription complex binding to enzalutamide-inducible genes may enhance the cell growth inhibitory effect of enzalutamide. Given that enzalutamide induces *NR3C1(GR)* transcription and that GR antagonist mifepristone is a clinically applicable agent (42), we next examined the effect of mifepristone alone or in combination with enzalutamide/K7174 on LNCaP cell growth. While exposure to mifepristone alone caused a modest decrease in the growth of LNCaP, co-treatment with mifepristone and enzalutamide led to stronger cell growth inhibition compared with enzalutamide alone (Supplementary Figure S17), suggesting that mifepristone augments the antigrowth effect of enzalutamide. Importantly, co-treatment of K7174 and enzalutamide showed stronger effect in inhibiting cell growth than combined treatment with mifepristone and enzalutamide (Supplementary Figure S17). In addition, there was no significant difference in cell growth inhibition between the mifepristone/enzalutamide/K7174 group and the enzalutamide/K7174 group (Supplementary Figure S17). These results suggest that targeting the GR upstream regulator GATA2 by K7174 is sufficient to inhibit enzalutamide-induced oncogenic transcription and thus sensitize prostate cancer cells to enzalutamide treatment.

## DISCUSSION

Although enzalutamide was initially thought to act as a pure AR antagonist (9), our recent finding that enzalutamide switches AR binding from canonical ARE to a distinct DNA motif (16) suggests that enzalutamide may act as a partial agonist in AR-mediated transcription. Indeed, our RNA-seq analysis found that enzalutamide stimulates a distinct class of cancer-relevant genes not affected by DHT (Figures 1, 2E and Supplementary Figure S1, 10). While the downregulation of some growth related genes such as *BIRC5* and *CDK1* by enzalutamide (Supplementary Figures S1 and S2) may account for enzalutamide-induced growth inhibition of prostate cancer cells (9,16), the upregulation of cancer-promoting genes such as *NR3C1* (23,43), *SLC7A11* (24–26) and *LAMP3* (44) by enzalutamide-liganded AR (Figure 1, Supplementary Figures S1, S3 and S10) suggests that these pathways may contribute to the emergence of resistance and that an opportunity exists to improve the therapeutic efficacy of enzalutamide by blocking these effects simultaneously.

GATA2 is the most highly expressed member of the GATA family in prostate tissue and is involved in prostate cancer aggressiveness (45). In prostate cancer, GATA2 facilitates androgen-responsive gene expression by three distinct modes of action: enhancing expression of AR itself, facilitating AR-enhancer binding by establishing an accessible local chromatin environment, and enhancing AR target gene expression through involvement in the formation and maintenance of regulatory chromatin loops between AR-bound distal enhancers and AR target gene promoters (21,46). GATA2 also regulates important non-androgen responsive genes (e.g. *IGF2*) involved in prostate cancer progression (47). Our results suggest that GATA2 directs enzalutamide-induced transcription by recruiting AR, MED1/MED14, and Pol II to regulatory elements of enzalutamide-responsive genes, revealing a novel role of GATA2 in directing antagonist-mediated transcription in prostate cancer. Previous studies have found that enzalutamide-responsive AR binding locations show higher chromatin accessibility than androgen-responsive AR binding locations (16). It is possible that GATA2, a transcription factor belonging to the GATA family that contains two zinc motifs mediating both chromatin binding and opening of the compact chromatin (48,49), contributes to the establishment of the highly accessible chromatin state on enzalutamide-responsive regions. Enzalutamide may also promote the formation of specific epigenetic architecture that facilitates GATA2 binding to enzalutamide-responsive regions (Figure 2A and Supplementary Figure S10D), resulting in a more open chromatin state. Future studies will be needed to test these hypotheses.

Although transcription factors are generally considered difficult to target in cancer therapy, the small molecule inhibitor K7174 has been used to target GATA2 regulation of androgen-responsive genes such as *PSA* and *TMPRSS2* (41). While a previous study shows that 20 μM K7174 decreases the GATA2 protein level and thus impairs GATA2-mediated transcription and prostate cancer growth (41), we found that 10 μM K7174 significantly decreases GATA2 chromatin binding and enzalutamide-induced transcription without affecting GATA2 protein levels (Figure 4). Since GATA2 directs enzalutamide-induced AR transcription complex binding (Figures 2, 3 and Supplementary Figure S10), it is very likely that inhibition of GATA2 binding by K7174 leads to impaired AR, Mediator and Pol II binding to enzalutamide-responsive gene loci (Figure 4 and Supplementary Figure S15). Importantly, the ability of K7174 to sensitize prostate cancer cells to enzalutamide treatment indicates that future studies should consider combining GATA2 inhibitors and enzalutamide to improve the therapeutic efficacy of enzalutamide in the treatment of prostate cancer.

It is worth noting that Shah *et al.* recently reported that AR binds to the –13.9 kb distal *NR3C1* enhancer in primary prostate cancer patient tissues (50). In CRPC cell models, they show that AR binds to this enhancer in the absence of ligand and that exposure to enzalutamide decreases AR binding, supporting their notion that AR binding to this enhancer represses *NR3C1* expression in CRPC cells, and that enzalutamide treatment decreases AR binding and thus releases this repression (i.e. de-repression) (50). In our studies, we find that AR binds to the same *NR3C1* distal element (Supplementary Figure S18) in the metastatic ADPC cell line LNCaP. Importantly, several lines of evidence support that this distal element acts as an active enhancer that drives enzalutamide-induced transcription in ADPC cells (Figure 1E–I, Figure 2D and Supplementary Figure S5). First, AR and Pol II binding as well as enrichment of active histone marks H3K4me1 and H3K27Ac on this distal region are augmented by enzalutamide treatment. Second, enzalutamide enhances the chromatin looping between this distal element and the *NR3C1* promoter. Third and most importantly, deletion of this distal element completely abolishes enzalutamide-induced transcription. Our findings thus reveal a novel active function of this enhancer in ADPC. Nonetheless, regardless of activation or de-repression of *NR3C1* expression by enzalutamide-liganded AR binding to this enhancer, GR expression is increased by enzalutamide treatment. Thus, GR inhibition decreases ADPC and CRPC prostate cancer cell growth and enhances the cell growth inhibitory effect of enzalutamide (Figure 4H, I, Supplementary Figure S11, S17) ((23,43,51,52). Importantly, since GATA2 functions upstream of *NR3C1* and other enzalutamide-induced cancer-promoting genes (e.g. *SLC7A11* and *LAMP3*) (Figures 2–4, Supplementary Figures S10, S13–S15), targeting GATA2 maybe a better strategy for improving AR-targeted therapy by enzalutamide (Supplementary Figure S17).

## DATA AVAILABILITY

The RNA-seq data has been deposited in the GEO database under the access code GSE125014.

## Supplementary Material

gkz790_Supplemental_File
