# Supplementary material for: Molecular determinants for enzalutamide-induced transcription in prostate cancer
Source: Nucleic Acids Res. 2019 Sep 10;47(19):10104–14. doi: 10.1093/nar/gkz790 (PMC6821169; doi:10.1093/nar/gkz790)
Supplement: gkz790_Supplemental_File [file gkz790_supplemental_file.doc]

**Molecular determinants for enzalutamide-induced transcription in prostate cancer**

Fuwen Yuan^1^**,** William Hankey^1^, Dayong Wu^2^, Hongyan Wang^1^, Jason Somarelli^3^, Andrew J. Armstrong^3,4,5^, Jiaoti Huang^1,4^, Zhong Chen^1^* & Qianben Wang^1,4^**

Supplementary Figures S1-S18

Supplementary Tables S1-S7

**SUPPLEMENTARY FIGURES**

Figure S1. Analysis of enzalutamide-regulated genes. (A) Unsupervised hierarchical clustering of enzalutamide-regulated genes (rows) from LNCaP cells in indicated conditions (columns) was performed. Red and green colors represent upregulation and downregulation, respectively. (B) A pie chart shows that 83 out of 91 enzalutamide upregulated genes (Fold>2, *p*<0.05) are not regulated by DHT. (C) Pathways and networks constructed using enzalutamide upregulated genes. Nodes and corresponding pathways are extracted according to interaction annotation.

Figure S2. Analysis of DHT-regulated genes. (A) Unsupervised hierarchical clustering of DHT-regulated genes (rows) from LNCaP cells under indicated conditions (columns) was conducted. Red and green colors represent upregulation and downregulation, respectively. (B) Pie charts show DHT-regulated genes also regulated by enzalutamide (13 enzalutamide upregulated genes and 46 enzalutamide downregulated genes). (C) Enriched pathways containing DHT-regulated genes.

Figure S3. Enzalutamide-liganded AR binding regulates distinct target genes compared with DHT-liganded AR. (A) LNCaP cells were cultured in 10% cFBS for 48 h, then vehicle or 10 nM DHT was added and cells were cultured for another 4 h or 24 h separately. Finally, total RNA was isolated for quantitative RT-PCR analysis. (B) LNCaP cells were cultured in 10% cFBS for 48 h, and treated with 25 µM enzalutamide for 4-48 h. Whole cell lysate was used for western blot analysis. (C) LNCaP cells were treated with vehicle or different doses of enzalutamide for 4 h or 24 h following growth in 10% cFBS for 48 h. Total RNA was isolated for quantitative RT-PCR analysis of the relative expression of *PSA* and *TMPRSS2*. (D and E) LAPC4 and CWR22Rv1 cells were cultured in 10% cFBS for 48 h and were treated with vehicle or 25 µM enzalutamide separately for 4 h or 24 h, then the total RNA was isolated and applied to quantitative RT-PCR analysis. (F) LNCaP cells were cultured in 10% cFBS for 48 h before treatment with vehicle or 10 µM or 25 µM enzalutamide for another 4 h, then cells were harvested for ChIP analysis using AR antibody. **p*<0.05, ***p*<0.01, results of ChIP and quantitative RT-PCR assays reported as the mean of two to four replicates, with error bars representing the standard deviation.

Figure S4. Treatment with bicalutamide or darolutamide but not apalutamide upregulates *NR3C1* and *SLC7A11* expression. (A-C) Expression of *NR3C1* and *SLC7A11* was determined by quantitative RT-PCR after LNCaP cells were treated with vehicle or 25 µM of bicalutamide (A), darolutamide (B) or apalutamide (C) for 4 h or 24 h. ***p*<0.01, ns, not significant, results of quantitative RT-PCR assays reported as the mean of two to four replicates, with error bars representing the standard deviation.

Figure S5. Distal AR binding elements are critical for enzalutamide induced *NR3C1* and *SLC7A11* transcription activation. (A) 3C analysis of interaction between the AR binding site and the *NR3C1* promoter region. LNCaP cells were grown for 72 h in the absence of hormone and then treated with vehicle or 25 µM enzalutamide for an additional 4h before cell harvest. 3C assays were performed using locus-specific primers. The grey shading indicates the AR binding fragment, while the black shading shows the *NR3C1* promoter position. (B and C) Top, gel images showing PCR amplification of genomic DNA using primers outside and inside of the enhancer region. sg-Control, a pair of sgRNAs that are predicted to not recognize any genomic regions; sg-del 1 and sg-del 2, two separate pairs of sgRNAs recognizing the boundaries of the *NR3C1* (B) and *SLC7A11* (C) enhancer regions. Bottom, PCR products were cloned into individual vectors and sequenced. Sequencing results represent the deletions induced by *NR3C1* sg-del 1 and sg-del 2 (B) and *SLC7A11* sg-del 1 and sg-del 2 (C). **p<0.01.

Figure S6. Heat map showing GATA2 ChIP-seq tag distribution in LNCaP cells around enzalutamide responsive AR binding locations within ± 2 kb.

Figure S7. The role of FOXA1 in enzalutamide-induced gene transcription. (A) LNCaP cells growing in 10% cFBS for 72 h were treated with vehicle or 25 µM enzalutamide for 4 h, then the cells were harvested for ChIP analysis of the enrichment of FOXA1 in the enhancer regions of representative genes. (B and C) Quantitative RT-PCR and western blotting were performed to determine the knockdown efficiency of *GATA2* and *FOXA1* after LNCaP cells were transfected with control siRNA or *GATA2* or *FOXA1* siRNA pool for 72 h. (D) LNCaP cells were transfected with control siRNA or *FOXA1* siRNA pool and cultured in 10% cFBS for 48 h, then cells were treated with vehicle or 25 µM enzalutamide for another 4 h or 24 h. Total RNA was then isolated to analyze the relative expression of representative genes using quantitative RT-PCR. **p*<0.05, ***p*<0.01, results of ChIP and quantitative RT-PCR assays reported as the mean of two to four replicates, with error bars representing the standard deviation.

Figure S8. (A) RNA-seq analysis of 25 µM enzalutamide and GATA2 knockdown-regulated genes. Unsupervised hierarchical clustering of genes (rows) regulated by 25 µM enzalutamide treatment and/or GATA2 knockdown- of LNCaP cells under indicated conditions (columns) was conducted. Red and green colors represent upregulation and downregulation, respectively. (B) Pie charts show enzalutamide-upregulated genes also upregulated by GATA2 (235 enzalutamide upregulated genes and 221 GATA2-upregulated genes).

Figure S9. The distribution of enzalutamide-liganded AR binding around the transcription start sites (TSS) of genes regulated by both enzalutamide and GATA2.

Figure S10. GATA2 knockdown inhibits enzalutamide-induced gene transcription. (A) LNCaP cells were transfected with *GATA2* siRNA or control siRNA, and cells were treated with vehicle or 25 µM enzalutamide for 24 h. Total RNA was isolated and amplified with gene-specific primers. (B) LNCaP cells were exposed to vehicle or 10 nM DHT for 24 h and total RNA was isolated and amplified with gene-specific primers. (C and D) AR (C) and GATA2 (D) LNCaP cells were transfected with GATA2 siRNA and/or treated with 25 µM enzalutamide, followed by ChIP assays to determine the relative enrichment of AR and GATA2 on distal regulatory regions from representative genes. Statistical significance was assessed using the Student’s *t*-test, ***p*<0.01, and results were reported as the mean of two to four replicates, with error bars representing the standard deviation.

Figure S11. Knockdown of enzalutamide-upregulated genes sensitizes LNCaP cells to enzalutamide-induced inhibition of cell proliferation. LNCaP cells were transfected with siCtrl (Control siRNA) or siRNA pools targeting the indicated genes. 24 h later, cells were split into 96 well plates (200~5000 cells per well) and treated with vehicle or 25 µM enzalutamide. WST-1 assays were performed to analyze the relative cell proliferation after 24 h, 72 h and 120 h, separately. ***p*<0.01, results reported as the mean of six replicates, with error bars representing the standard deviation.

Figure S12. *MED1* and *MED14* coactivate enzalutamide-induced transcription. (A and B) Quantitative RT-PCR (A) and western blot (B) assays were performed after LNCaP cells were transfected with siMED1 or siMED14. (C) Total RNA was isolated to analyze the relative expression of representative genes using quantitative RT-PCR after LNCaP cells were transfected with control siRNA or *MED1* or *MED14* siRNA pools and then treated with vehicle or 25 µM enzalutamide for the indicated time. ***p*<0.01, results of RT-PCR assays reported as the mean of two to four replicates, with error bars representing the standard deviation.

Figure S13. LNCaP cells were treated for 24 h with vehicle, 25 µM enzalutamide, 10 µM K7174 or enzalutamide together with K7174, and total RNA was subjected to RT-PCR to analyze relative gene expression. ***p*<0.01. Results of quantitative RT-PCR assays are reported as the mean of two to four replicates, with error bars representing the standard deviation.

Figure S14. GATA2 inhibitor K7174 disturbs enzalutamide-induced gene transcription in LAPC4 cells . (A) LAPC4 cells were cultured in 10% cFBS for 48 h and were treated with vehicle or 25 µM enzalutamide or 10 µM K7174 or 25 µM enzalutamide together with 10 µM K7174 for 24 h. Quantitative RT-PCR was performed with the total RNA. (B) LAPC4 cells were treated with vehicle or 10 µM K7174 for 24 h. Then cells were harvested, and total protein was applied for western blot analysis using the indicated antibodies. ***p*<0.01. Results of quantitative RT-PCR assays are reported as the mean of two to four replicates, with error bars representing the standard deviation.

Figure S15. GATA2 inhibitor K7174 attenuates enzalutamide-induced gene transcription. LNCaP cells were cultured in charcoal-stripped FBS for 48 h, treated with vehicle or 10 µM K7174 for 20 h, then treated with vehicle or 25 µM enzalutamide for another 4 h. AR, MED1 and MED14 ChIP assays were performed with detection of distal *TSC22D3, LAMP3, VEGFA* and *CEMIP* elements, and Pol II ChIP assays were conducted with detection of the *TSC22D3, LAMP3, VEGFA* and *CEMIP* promoters. ***p*<0.01. Results of ChIP assays are reported as the mean of two to four replicates, with error bars representing the standard deviation.

Figure S16. GATA2 inhibitor K7174 disturbs enzalutamide-induced gene transcription and decreases LAPC4 cell proliferation. LAPC4 cells were split in 96 well plates (5000 cells per well) and were treated with vehicle or 10 µM K7174 together with different concentrations of enzalutamide for 72 h. WST-1 assays were performed to analyze relative cell proliferation. ***p*<0.01, results of WST-1 assays reported as the mean of two to six replicates, with error bars representing the standard deviation.

Figure S17. K7174 together with enzalutamide shows the strongest combined cell growth inhibition effect in LNCaP cells. LNCaP cells were split in 96 well plates (5000 cells per well) and were treated with vehicle, 25 µM enzalutamide, 10 µM K7174, 100 nM mifepristone, or a combination of enzalutamide with a second chemical for 72 h. BrdU incorporation assays were performed to analyze relative cell proliferation. (**p<0.01, ns, not significant, results of BrdU incorporation assays reported as the mean of six replicates, with error bars representing the standard deviation.

Figure S18: UCSC Genome Browser views of the distribution of H3K27ac and H3K4me1 in LNAR, LREX, LREXEnz cells, as well as AR binding in primary prostate cancer tissues (1). and both H3K4me1/2 and AR binding in LNCaP cells at the *NR3C1* locus (2).

**Table S1 siRNA sequences**

| siGATA2 (Dharmacon ON TARGET plus siRNA) | (1) UCGAGGAGCUGUCAAAGUG  (2) ACUACAAGCUGCACAAUGU  (3) GAAGAGCCGGCACCUGUUG  (4) GCCCAGGCCUAGCUACUAU |
| --- | --- |
| siFOXA1 (Dharmacon ON TARGET plus siRNA) | (1) GCACUGCAAUACUCGCCUU  (2) CCUCGGAGCAGCAGCAUAA  (3) CCUAAACACUUCCUAGCUC  (4) GAACAGCUACUACGCAGAC |
| siMED1 (Dharmacon ON TARGET plus siRNA) | (1) GCAGAGAAAUCUUAUCAGA  (2) CCAUUAAGCUUGUGCGUCA  (3) CAGCAAUGACUGAUCGUUU  (4) GGCCGAAGAGCAAGGCUUA |
| siMED14 (Dharmacon ON TARGET plus siRNA) | (1) GAAUAGCAUGCACGAUUA  (2) CCACACAACUGUCGUACAA  (3) GCACUUGGGUAGCAGAGUU  (4) GGAUGCAAUUCGCUUAUUA |
| Non-targeting pool (Dharmacon ON TARGET plus siRNA) | (1) UGGUUUACAUGUCGACUAA  (2) UGGUUUACAUGUUGUGUGA  (3) UGGUUUACAUGUUUUCUGA  (4) UGGUUUACAUGUUUUCCUA |

**Table S2 10 µM Enzalutamide upregulated genes**

| CD55 | KLHDC7B | FOCAD-AS1 |
| --- | --- | --- |
| TRIB3 | F3 | ADRA1A |
| CHAC1 | DIO3 | PXDC1 |
| SLC6A9 | FAM84A | LTF |
| TENM1 | FGFR2 | MCHR2 |
| TSC22D3 | PDE2A | C1orf220 |
| FAM129A | SLC7A11 | SLURP1 |
| C1orf158 | FRG1DP | ATE1-AS1 |
| CCPG1 | STKLD1 | TGM1 |
| CAPN5 | TSLP | TMPRSS3 |
| DDIT3 | COLCA2 | PLAC1 |
| DMGDH | FAM167A | CEMIP |
| HIST1H4D | FLRT1 | LURAP1L |
| FAM131B | SPX | LOC101928020 |
| ADM2 | SERPINA3 | C20orf197 |
| CDH26 | COL16A1 | DGCR5 |
| DNAH10 | BARX1 | WNT5B |
| BCAT1 | FAM225A | GPAT3 |
| ASNSP1 | GPR1 | DNER |
| FIGNL2 | VLDLR-AS1 | LCN2 |
| BEST1 | BCL11B | SCARNA7 |
| FIBIN | FLJ36000 | LOC101928605 |
| NR3C1 | PABPC1L | LOC284577 |
| KRT75 | ARHGAP9 | ARHGAP19-SLIT1 |
| ETV5 | LINC01277 | LOC102724450 |
| PLEKHH2 | PIK3R6 | RRAD |
| INHBE | ANGPT2 | STARD4-AS1 |
| DHRS3 | RELN | LOC105377763 |
| LINC01512 | RORA | MBD3L2B |
| MOV10L1 | PCOLCE2 |  |
| SGK2 | PHF21B |  |

**Table S3** 25 µM Enzalutamide upregulated genes

| SNORA44 | VLDLR-AS1 | GPR1 | HMOX1 |
| --- | --- | --- | --- |
| LAMP3 | PDE2A | PTHLH | RNASEK-C17orf49 |
| OLIG2 | GOLGA8H | GCNT3 | SLC43A3 |
| FGG | WNT11 | CHRFAM7A | FOS |
| PRSS3P2 | CAMP | RNF219-AS1 | KCNA4 |
| FIBIN | PTGER1 | CAPN5 | MAGEL2 |
| FCRLA | STT3A-AS1 | KLHDC7B | CCDC180 |
| SEPT7P9 | SERPINA3 | LOC100505501 | DIO3 |
| CHST8 | MRVI1-AS1 | TRIB3 | CHRNA7 |
| UBE2QL1 | ACTR3-AS1 | TMIE | LINC01133 |
| FLRT1 | FAM129A | LINC00365 | FTH1 |
| TMEFF1 | TM6SF1 | PIP5KL1 | FAM167A |
| CYP2B7P | DDIT3 | CD55 | TMEM89 |
| HABP2 | LOC101929224 | FAM225A | SERPINF1 |
| MOV10L1 | ALDH3A1 | CA7 | TSC22D3 |
| C5orf49 | CES5A | UGT1A6 | C10orf67 |
| HSD17B2 | NGFR | LOC102723692 | ASNS |
| FGF21 | FAM225B | LOC101927989 | CEMIP |
| PSMB11 | MYHAS | USP30-AS1 | SULT1C2 |
| C1orf158 | SNORD27 | PSD2 | PCK2 |
| CHAC1 | SPX | LGALS4 | MRGPRE |
| LPO | PRSS3 | PIP5K1B | CDHR2 |
| DMGDH | CHRDL2 | DDIT4 | ID4 |
| FGF19 | RGS7BP | HOTS | RHCE |
| PRSS1 | KCNK15-AS1 | ZDHHC19 | GDF15 |
| S100P | TGM1 | INHBE | ERICH2 |
| LINC01512 | GPM6A | ASNSP1 | LGR6 |
| CALCA | NR3C1 | TCTE1 | GRM4 |
| LINC02485 | LOC100507006 | IL2RB | DNAH10 |
| SCRG1 | ZNF32-AS3 | LOC101928358 | TMEM178A |
| SULT4A1 | TTC39B | CCL22 | SLC3A2 |
| CALCB | TTC39C-AS1 | TMEM105 | ENTPD3 |
| ETV5 | CDKN2B | NR1D1 | LINC00900 |
| FTH1P3 | CYP4F3 | ZNF32-AS2 | SLC16A1-AS1 |
| DUSP15 | NUPR1 | UPP1 | PHF24 |
| ATF3 | SYCP2L | NTN4 | FYN |
| ERN1 | SLC6A9 | HHIPL1 | ATP2C2 |
| LOC105372273 | PRDM16 | WEE2 | BGN |
| PANX3 | RNF144B | BBC3 | SNORA21 |
| RAB38 | EGF | IDH1 | PRKN |
| LOC100996251 | ZNF423 | MOCOS | IFRD1 |
| CTH | PSMB9 | FSIP1 | BCL11B |
| FOCAD-AS1 | ELF3 | AKR1C2 | CNGA1 |
| STC2 | SLC1A4 | SLC7A5 | COL16A1 |
| RELN | F7 | MMP28 | IRF1 |
| TRIM31 | VEGFA | GAD1 | MCF2L2 |
| SP7 | ADRA1A | DNM1P35 | CTAGE11P |
| FOXJ1 | NPIPB11 | TRIM49B | TGM6 |
| DHRS3 | MAL | PAK6 | FIGNL2 |
| SLC7A11 | NUCB2 | LAMB2P1 | FRAS1 |
| PLIN1 | SARS | ODF3B | OSGIN1 |
| CEBPB | CZ1P-ASNS | ZNF503-AS1 | SLC1A5 |
| ST6GALNAC2 | TBX2-AS1 | ANK2 | CNTFR |
| CSTA | LOC100506544 | ADM2 | TUBB2B |
| SVIL | ALDH1L2 | AGBL4 | NKX2-8 |
| KLF3-AS1 | IQCJ-SCHIP1 | BEAN1 |  |
| ATF4 | NMRAL2P | PSAT1 |  |
| LINC00689 | MIA2 | CARD9 |  |
| HCK | PLEKHH2 | ULBP1 |  |
| PRSS33 | ZNF575 | PIK3R6 |  |

**Table S4** ChIP PCR primers

| NR3C1 Enhancer | Forward | ACGTGCTCTTTAACCAGCAAAAT |
| --- | --- | --- |
|  | Reverse | GTGTGGAGAACCCAACTAGGA |
| SLC7A11 Enhancer | Forward | CTGGCGCTGCTTGTGAAAAT |
|  | Reverse | GGTCTGCAAGTGGTGAAAGG |
| NR3C1 Promoter | Forward | CACGTTCATAGGACCCCTGC |
|  | Reverse | TCCTTTCTCAGGACGGACCA |
| SLC7A11 Promoter | Forward | ACCGTCCAGATGGTCAGAGA |
|  | Reverse | TTGGCACCATCATTGGAGCA |
| PSA Enhancer | Forward | TGGGACAACTTGCAAACCTG |
|  | Reverse | CCAGAGTAGGTCTGTTTTCAATCCA |
| TMPRSS2 Enhancer | Forward | TGGTCCTGGATGATAAAAAAAGTTT |
|  | Reverse | GACATACGCCCCACAACAGA |
| TSC22D3 Promoter | Forward | TCTCCGTCTGTCGTTACCCT |
|  | Reverse | GAAACCCTGGAAACCCACCA |
| LAMP3 Promoter | Forward | AGAGAAACCTACCTGTGCCG |
|  | Reverse | GTGCGGTAAACGGTCCCA |
| VEGFA Promoter | Forward | TAGCAAAGAGGGAACGGCTC |
|  | Reverse | AACTCTGTCCAGAGACACGC |
| CEMIP Promoter | Forward | GGAGGGAAGTTTCATCGGGG |
|  | Reverse | AGAGGCCGCTTTTATAGCCA |
| TSC22D3 Enhancer | Forward | CTTTACAAGGGACCACGTCAAT |
|  | Reverse | AAGCAATTTGAGGGTAGGGC |
| LAMP3 Enhancer | Forward | CAGCCTGGCCAGTATGTACC |
|  | Reverse | TCTATGTGCAGGCACTGACG |
| VEGFA Enhancer | Forward | GCCATTCACAGTATTCTGCACC |
|  | Reverse | GGAAGCTCTTGCTGCTCATGG |
| CEMIP Enhancer | Forward | CCCGCCCGGGACAATATTTA |
|  | Reverse | GAACCGCCTGGCTCAGTAG |

**Table S5** Quantitative RT-PCR primers

| 18S rRNA | Forward | GTAACCCGTTGAACCCCATT |
| --- | --- | --- |
|  | Reverse | CTACCACATCCAAGGAAGCA |
| NR3C1 | Forward | GCAAAGGCAATACCAGGTTTCA |
|  | Reverse | CAGGAGCAAAACACAGCAGG |
| SLC7A11 | Forward | ATGCAGTGGCAGTGACCTTT |
|  | Reverse | CATGGAGCCAAAGCAGGAGA |
| PSA | Forward | ACGCTGGACAGGGGGCAAAAG |
|  | Reverse | GGGCAGGGCACATGGTTCACT |
| TMPRSS2 | Forward | GGACAGTGTGCACCTCAAAGAC |
|  | Reverse | TCCCACGAGGAAGGTCCC |
| GATA2 | Forward | AGGAAGGGATCCAGACTCGG |
|  | Reverse | TTTGACAGCTCCTCGAAGCA |
| FOXA1 | Forward | ACAGCTACTACGCAGACACG |
|  | Reverse | CCCAGGCCTGAGTTCATGTT |
| MED1 | Forward | TGGACTGGGCTCTCATCTCA |
|  | Reverse | TAGCTGCTGTACAAGCTCCG |
| MED14 | Forward | GGCTGAGCACCCTCATTGAA |
|  | Reverse | TTGGCAGTAGGTCCGTCAAC |
| TSC22D3 | Forward | GCAGACCGCTAACTAGCTCA |
|  | Reverse | AGGGTGCCTGGAAAAGACAA |
| LAMP3 | Forward | CCTGCAGGTGAAAACAACCG |
|  | Reverse | ATGGCCCCAATCACAGGAAG |
| VEGFA | Forward | AAGGGGCAAAAACGAAAGCG |
|  | Reverse | GGAGGCTCCAGGGCATTAGA |
| CEMIP | Forward | TTCAAGACGTGGAGTGGACG |
|  | Reverse | CTGGGTGAGCTTTCCAGAGG |

**Table S6 3C primers (Sequence (5’-3’))**

| NR3C1-Region1 | TGAGACAGGATTTGACCCACT |
| --- | --- |
| NR3C1-Enhancer | TGTTTAGTGTGTGGAGAACCC |
| NR3C1-Region3 | AGTCAAGTCTAGGAGCAGCCC |
| NR3C1-Region4 | ACAGTGGGGTTTCTTATAAATGCAC |
| NR3C1-Region5 | CTGCCCATTGCACTGATTCC |
| NR3C1-Region6 | GAGTTAGCCTGCCCAAGGTT |
| NR3C1-anchor | ACTTTGACATCAACTTGAACCT |
| NR3C1-Tagnan probe | CTATTCATCCTGCCGCTCACTGAAC |
| NR3C1-Region7 | ACGTTCAAATGTTCCTGCAAGTT |
| NR3C1-Region8 | ATGGCATTAACTTAGCACTGGGA |
| NR3C1-Region9 | GCCTAATGTGAAAGTCGGAAC |
| NR3C1-Region10 | AACTCTGGTCTGTTTACCATTGA |
| NR3C1-Region11 | TTTGCAAATGCCCCTTCAGA |
| 3C GADPH loading control+ | ACAGTCCATGCCATCACTGCC |
| 3C GADPH loading control- | GCCTGCTTCACCACCTTCTTG |
| 3C GADPH Hind III control+ | CTAGGCAGCAGCAAGCATTC |
| 3C GADPH Hind III control- | TGAGCTGCTTCAACTTTGGAAT |

Table S7 Primers used for enhancer deletion

| Dummy_sg1_F | ATATCGTCTCACACCGGGCCCGCATAGGATATCGCGTTTTAGAGCTAGAAATAGCAAG |
| --- | --- |
| Scaffold_R | AATTCGTCTCGGCTAATGTAAAAAAGCACCGACTCGGTG |
| H1promoter_F | AATTCGTCTCTTAGCGCTTGAACGCTGACGTCATCAAC |
| Dummy_sg2_R | ATCGTCTCAAAACCGCCGTTAAGCGGAAACGATGGGAAAGAGTGGTCTCATACAGAACT |
| NR3C1del_sg1_F | ATATCGTCTCACACCAATGATCGGGTTTCCTCTTAGTTTTAGAGCTAGAAATAGCAAG |
| NR3C1del_sg2_R | ATCGTCTCAAAACATGGGCGTGTACTTTCTGCTGGGAAAGAGTGGTCTCATACAGAACT |
| NR3C1del_sg3_F | ATATCGTCTCACACCTAACAGAACTGGCTTGGACGGTTTTAGAGCTAGAAATAGCAAG |
| NR3C1del_sg4_R | ATCGTCTCAAAACCCTTACATCATGGATTGTGAGGGAAAGAGTGGTCTCATACAGAACT |
| SLC7A11del_sg1_F | ATATCGTCTCACACCCCCGGAAACCGTAGAACAGCGTTTTAGAGCTAGAAATAGCAAG |
| SLC7A11del_sg2_R | ATCGTCTCAAAACTCATAGGATGTAGGCAACTGGGGAAAGAGTGGTCTCATACAGAACT |
| SLC7A11del_sg3_F | ATATCGTCTCACACCACTCCTGCTCGTCAAGCTACGTTTTAGAGCTAGAAATAGCAAG |
| SLC7A11del_sg4_R | ATCGTCTCAAAACTTGATGAGACTATATTAGCCGGGAAAGAGTGGTCTCATACAGAACT |
| qNR3C1-F1 | GATAGCCCTGTAGGCACACC |
| qNR3C1-R1 | CTCATTGGTGACAACAGCCTTAAG |
| qNR3C1-F2 | TATCAGGCCTCTCCCAGCTT |
| qNR3C1-R2 | TTCACCTTCTGACCTGTGCC |
| SLC7A11-F1 | GGACTCATCCTCACAGAG |
| SLC7A11-R1 | AGACCAATGAGTTTTAATGCCG |
| SLC7A11-F2 | ATAAACATTGACTGGCGCTGC |
| SLC7A11-R2 | ATGTAGGCAACTGTGGTAACTCT |

**REFERENCES FOR SUPPLEMENTAL MATERIALS**

1. Shah, N., Wang, P., Wongvipat, J., Karthaus, W.R., Abida, W., Armenia, J., Rockowitz, S., Drier, Y., Bernstein, B.E., Long, H.W. *et al.* (2017) Regulation of the glucocorticoid receptor via a BET-dependent enhancer drives antiandrogen resistance in prostate cancer. *Elife*, **6,** e27861.

2. Chen, Z., Lan, X., Thomas-Ahner, J.M., Wu, D., Liu, X., Ye, Z., Wang, L., Sunkel, B., Grenade, C., Chen, J. *et al.* (2015) Agonist and antagonist switch DNA motifs recognized by human androgen receptor in prostate cancer. *EMBO J*, **34**, 502-516.
